# Cerebrovascular events, bleeding complications and device related thrombi in atrial fibrillation patients with chronic kidney disease and left atrial appendage closure with the WATCHMAN™ device

**DOI:** 10.1186/s12872-019-1097-0

**Published:** 2019-05-15

**Authors:** Blerim Luani, Conrad Genz, Joerg Herold, Andreas Mitrasch, Julius Mitusch, Marcus Wiemer, Alexander Schmeißer, Rüdiger C. Braun-Dullaeus, Thomas Rauwolf

**Affiliations:** 1 Department of Cardiology and Intensive Care Medicine, Johannes Wesling University Hospital, Ruhr University Bochum, Hans-Nolte-Str. 1, 32429 Minden, Germany; 2 Department of Internal Medicine, Division of Cardiology and Angiology, Magdeburg University, Leipzigerstr 44, 39120 Magdeburg, Germany

**Keywords:** LAA closure, Watchman device, Chronic kidney disease, Oral anticoagulation, Cerebrovascular events, Bleeding complications, Device related thrombi

## Abstract

**Background:**

Impaired renal function increases the bleeding risk, leading to a conservative prescription and frequent discontinuation of oral anticoagulation in atrial fibrillation patients with chronic kidney disease (CKD). Interventional left atrial appendage closure (LAAC) might be an alternative therapeutic strategy for these patients.

**Methods:**

We aimed to prospectively assess cerebrovascular (CE) and bleeding events, as well as peri-procedural and long-term complications in a cohort of consecutive patients undergoing interventional LAAC using the WATCHMAN™ device, with focus on CKD patients.

**Results:**

One hundred and eighty-nine consecutive patients undergoing interventional LAAC were included in this analysis; 171 (90.5%) patients had a reduced estimated glomerular filtration rate (eGFR; patients for each CKD stage: II = 66; IIIa = 32; IIIb = 43; IV = 18; V = 12). During a follow-up of 310 patient years three (1.0%) patients suffered a CE (two strokes, one transitory ischemic attack) and five (1.6%) other ones a bleeding complication. The observed stroke rate was more than two-thirds and the bleeding risk more than half lower than expected. Device related thrombi (DRT) were detected in twelve (6.5%) patients; women had significantly more DRT than men (12.5% vs. 2.6%; *p* = 0.009). Patients with an eGFR< 30 ml/min/1.73m^2^ showed a trend to a higher DRT rate as compared to the opposite group (13.3% vs. 5.1%; *p* = 0.10). Thrombus resolved with temporary oral anticoagulation therapy in ten patients without sequelae; thrombus consolidation was confirmed by serial TEE controls in the remaining two patients.

**Conclusions:**

Atrial fibrillation patients with CKD have low CE and bleeding rates after LAAC with the WATCHMAN™ device. DRT risk is higher in female and patients with severe CKD. More frequent post-interventional TEE controls might be justified for early DRT detection and safe management of patients at high DRT risk.

**Trial registration:**

(German Clinical Trials Register ID: DRKS00 010768; Registration Date 07.07.2016).

## Background

Non-valvular atrial fibrillation (NVAF) is the most common cardiac arrhythmia, which is associated with an increased morbidity and mortality, mainly due to a higher risk of stroke [1]. The pathomechanism of the increased stroke risk in NVAF patients is thought to be the embolism of intracardiac thrombi, which are far more frequent in these patients and mostly found in the left atrial appendage (LAA) [2]. A major corner stone in the management of NVAF patients at moderate or high risk of thromboembolic complications is the preventive oral anticoagulation (OAC) therapy tailored on a risk to benefit relation, which can both be estimated by appropriate risk scores (e.g. CHA_2_DS_2_Vasc and HAS-BLED scores) [3, 4]. Over the past decades vitamin K antagonists (VKA) were used as the gold standard medication for this purpose, resulting in a significant reduction of the stroke risk and mortality (by approximately two thirds and one fourth to one third, respectively) [5]. The medication with the relatively new non-vitamin-K antagonist oral anticoagulants (NOACs) showed a more favorable profile with a further reduction of stroke risk, as compared to VKA [6–10]. Considering the actual data, the current guidelines recommend a preventive oral anticoagulation therapy (preferably NOACs) in patients with NVAF and a CHA_2_DS_2_-VASc-Score of ≥1 (≥ 2 in women) [11]. However, several shortcomings of this therapy have been reported; at the time of VKAs being the only treatment option, just about half of the NVAF patients eligible for OAC therapy did receive a VKA [12] and in those treated with a VKA, the time within the standard target therapeutic range (INR 2.0–3.0) was just about two thirds of the total treatment time despite frequent monitoring [13]. Undertreatment remains an important issue in the NOAC’s era, as well, with a drug discontinuation rate of up to 34%, even in the setting of large randomized trials [6–9]. Furthermore, NOAC therapy is not recommended in stage V chronic kidney disease (CKD) patients and the benefit of VKA-therapy has not been unambiguously demonstrated in that cohort [14]. Since NVAF patients with CKD are at increased risk of both stroke and bleeding complications [15–19], the net clinical benefit of the OAC therapy in these patients needs to be carefully evaluated. LAA closure is an alternative therapeutic strategy to a lifelong anticoagulation in NVAF patients with moderate or high thromboembolic risk and a high bleeding risk or bleeding history [20–23], but data on short- and long-term efficacy or safety of interventional LAA-closure in patients with CKD are scarce.

We aimed to prospectively assess thromboembolic and bleeding events as well as periprocedural and long-term complications in a cohort of consecutive atrial fibrillation patients undergoing interventional closure of the LAA using the WATCHMAN™ device with focus on CKD patients.

## Methods

**Fig. 1:**
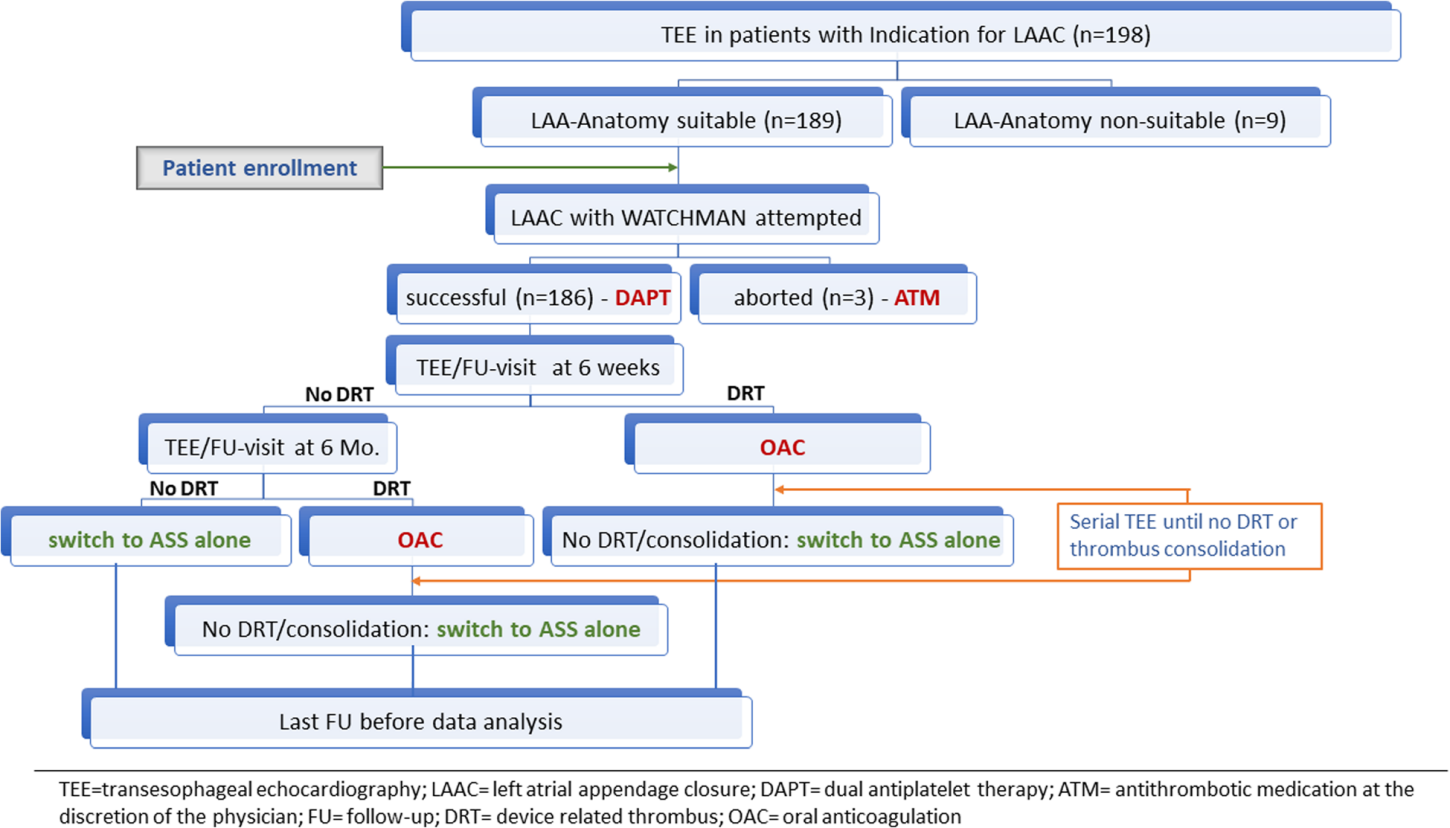
Flowchart of the inclusion, management and follow-up of the patients

Consecutive NVAF patients with clinical indication and suitable anatomy for interventional LAA closure, as assessed by TEE prior to the implantation procedure, were included in this study after giving their written informed consent; Fig. 1 shows a flowchart of patient inclusion, management and follow up. A diagnostic evaluation including inquiry of medical and drug history, physical examination and routine blood tests was accomplished before the implantation procedure. Effective LAA closure (defined as no or residual leakage < 5 mm between the occluder device and the LAA wall), potential device dislocation or embolization and device related thrombi (DRT) were assessed by transesophageal echocardiography (TEE) at the index procedure and the scheduled controls after six weeks and six months. Clinical safety and efficacy data after successful LAA occlusion were collected during the in-hospital treatment, at the above-mentioned outpatient visits and by phone contact prior to the final data analysis. Clinical efficacy data included rates of cerebrovascular events or peripheral embolism, whereas safety data included periprocedural complications, potential device dislocation or embolization, DRT and bleeding events; intracranial bleeding or those requiring transfusion were classified as major ones. In cases with thromboembolic or bleeding complications reports from the respective treating center were collected.

Renal function of the study subjects was classified according to the Kidney Disease Improving Global Outcomes (KDIGO) 2017 clinical practice guideline update [24]. The estimated glomerular filtration (eGFR) rate was calculated using the Chronic Kidney Disease Epidemiology Collaboration (CKD-EPI) formula at the time of hospital admission for device implantation [25]. The study was approved by the local Ethics Committee.

### WATCHMAN™ device and implantation procedure

Interventional LAA closure using a 3rd (or 4th) generation WATCHMAN™ (WATCHMAN FLX™) device (Boston Scientific, Marlborough, USA) was performed under TEE and fluoroscopic guidance in our catheterization lab. We have described the device and implantation procedure previously in detail [26]. Shortly, the size and morphology of the LAA were assessed by TEE standard views and fluoroscopy using a pigtail diagnostic catheter for contrast media injection after gaining transseptal access. Device size was selected upon manufacturer recommendation on the compression ratio. The device was deployed only if a residual leakage was absent or < 5 mm. In patients with a special LAA anatomy with proximal arborization into two or more dominant lobes a two-device implantation strategy was considered.

The post-implantation dual antiplatelet therapy with clopidogrel and aspirin was limited to aspirin monotherapy, after DRT exclusion at the last scheduled TEE control (after six-months).

### Echocardiographic parameters

Performance of the occluder device regarding effective LAA closure (= residual leakage < 5 mm) and potential complications such as device dislocation, DRT formation, pericardial effusion, presence and severity of mitral valve regurgitation were assessed by transesophageal echocardiography at the index procedure and scheduled controls after six weeks and six months. Furthermore, maximum and minimal device compression (DC_max/min_) and compression rate (CR_max/min_) as assessed by TEE measurements at the index procedure were investigated as potential predictive parameters of residual leakage. DC_max_ was defined as the ratio between the nominal device size and minimal device diameter, whereas DC_min_ as the ratio between the nominal device size and maximal device diameter as measured by TEE after deployment. Similarly, CR_max_ was defined as the ratio between nominal device size and minimal LAA ostium diameter, whereas CR_min_ as the ratio between nominal device size and maximal LAA ostium diameter as measured by TEE in the standard views (45°, 60°, 90° and 135°) before device deployment.

### Statistics

All patients included in the registry were considered for analysis of the data. Categorical parameters are presented as counts and percentages, whereas continuous variables as mean values ± standard deviation (SD). Categorical variables were compared by the chi-square test. Continuous variables were compared using the matched-pairs t-test after controlling for normal distribution by the Kolmogorov-Smirnov test. Estimated annual stroke or bleeding risks according to the CHA2DS2Vasc and HAS-BLED scores, respectively, are based on data from Lip and colleagues [3, 4, 27]. Statistical calculations were performed using the dedicated software SPSS Version 18 (IBM, Armonk, NY, USA).

## Results

One hundred ninety-eight consecutive patients, who were assigned to interventional LAA closure in our catheterization laboratory, were included in this registry. Interventional LAA closure was not attempted in nine patients because of unsuitable anatomy (*n* = 6; usually very small LAA dimensions) or detected thrombus within the LAA (*n* = 3), as assessed by TEE prior to the procedure. In three more patients the procedure was aborted because of ineffective LAA closure (= residual leakage ≥5 mm). Consequently, successful device implantation with confirmation of effective LAA closure occurred in 186 (98.4%) patients.

The mean patient age was 73.4 ± 7.9 years (61.4% men). The indication for interventional LAA closure was previous bleeding event(s) in 108 (57.1%), increased bleeding risk (as assessed by a HAS-BLED score ≥ 3) in 58 (30.7%), patient preference in 6 (3.2%) and contraindication to oral anticoagulation therapy in 17 (9.0%) patients. From the 108 patients with a bleeding history, 61 (56.5%) patients had suffered a gastrointestinal bleeding, 12 (11.1%) patients an intracranial bleeding and 35 (32.4%) patients other bleeding complications such as hematuria, hemoptysis, intraocular bleeding etc. Mean CHA_2_DS_2−_Vasc and HAS-BLED score were 3.88 ± 1.6 and 3.35 ± 1.0, respectively. Moderate or severe impairment of renal function with an eGFR of less than 45 ml/min/1.73m^2^ was present in 73 (38.6%) patients (number of patients for each CKD stage: II = 66; IIIa = 32; IIIb = 43; IV = 18 and V = 12). The baseline characteristics of the analyzed patient population as well as of the subgroups with eGFR < 45 vs. ≥ 45 ml/min/1.73m^2^ are shown in Table 1. Patients with moderate or severe eGFR reduction (CKD IIIb or worse) suffered more often from diabetes mellitus (61.6% vs. 34.5%, *p* < 0.01), had a higher CHA_2_DS_2−_Vasc-score (4.50 ± 1.42 vs. 3.40 ± 1.57; p < 0.01) and a higher HAS-BLED score (3.65 ± 1.0 vs. 3.13 ± 0.9; p < 0.01) compared to the opposite group (CKD IIIa or better), see Table 1 for details.

**Table 1:** Patient baseline characteristics

	All patients	Pt. with eGFR≥45	Pt. with eGFR< 45	*p*
n	189	116	73	
Male sex, n (%)	116 (61.4)	74 (63.8)	42 (57.5)	0.18
Mean age (years)	73.4 ± 7.9	72.1 ± 8.0	75.9 ± 6.7	0.10
BMI (kg/m^2^)	29.3 ± 5.4	28.94 ± 5.5	29.28 ± 5.2	0.83
BSA (m^2^)	1.96 ± 0.23	1.96 ± 0.20	1.95 ± 0.24	0.58
CHA_2_DS_2_-VASc score	3.88 ± 1.6	3.40 ± 1.57	4.50 ± 1.42	< 0.01
HAS-BLED – Score	3.35 ± 1.0	3.13 ± 0.9	3.65 ± 1.0	< 0.01
Arterial hypertension, n (%)	164 (86.8)	98 (84.5)	66 (90.4)	0.32
Diabetes mellitus, n (%)	85 (45.0)	40 (34.5)	45 (61.6)	< 0.01
Hyperlipidaemia, n (%)	94 (49.7)	56 (48.3)	38 (52.1)	0.42
Smoking status				
non-smoker, n (%)	138 (73.0)	81 (69.8)	57 (78.1)	0.12
smoker, n (%)	25 (13.2)	15 (12.9)	10 (13.7)	0.70
stopped smoking, n (%)	26 (13.8)	20 (17.2)	6 (8.2)	0.10
Atrial fibrillation				
paroxysmal, n (%)	75 (39.7)	48 (41.4)	27 (37.0)	0.33
pers./perm., n (%)	114 (60.3)	68 (58.6)	46 (63.0)	
Previous stroke / TIA, n (%)	33 (17.2)	21 (17.5)	12 (16.4)	0.80

**Fig. 2:**
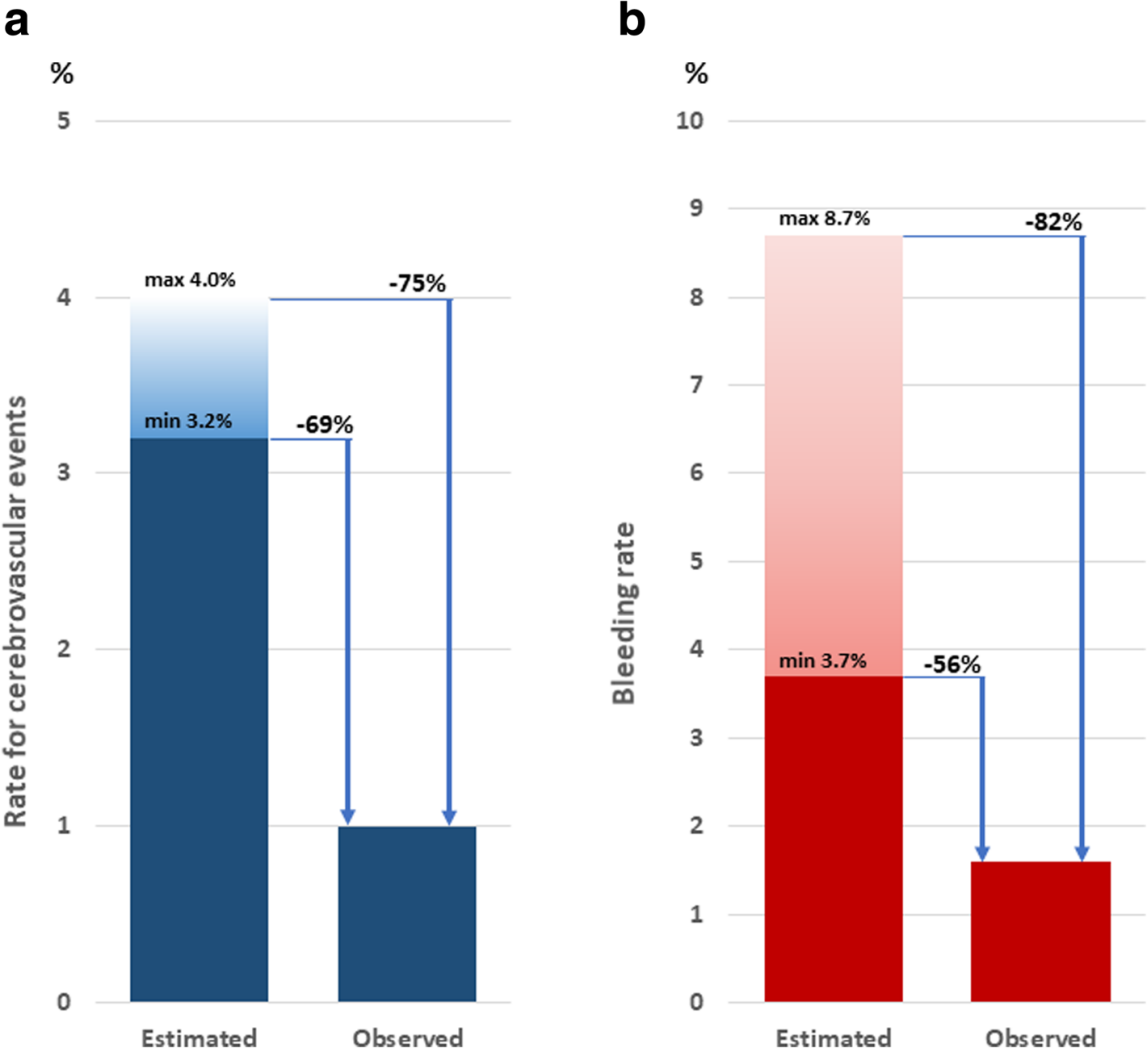
Estimated and observed rates of **a**) cerebrovascular events and **b**) bleeding complications in the study population

During a follow-up of 310 (1.6 ± 1.0) patient years three (1.0%) patients (all three with eGFR < 45 ml/min/1.73m^2^) suffered thromboembolic events including two ischemic strokes (one perioperative to a transcutaneous aortic valve replacement) and one transitory ischemic attack, see Table 2 for details. The estimated annual rate for cerebrovascular events according to a CHA2DS2Vasc score between 3 and 4 (3.88 in the analyzed population) ranged from 3.2 to 4.0%. The CHA2DS2Vasc-Score was higher in patients with reported thromboembolic events, as compared to those without (5.3 ± 0.5 vs. 3.7 ± 1.7, p = n.s.). Bleeding complications were observed in five (1.6%) patients, all with moderate or severe impairment of the renal function (eGFR < 45 ml/min/1.73m^2^); one patient suffered a gastrointestinal bleeding two days after a colonoscopy with polypectomy while being on aspirin monotherapy; in two other patients macrohematuria occurred, one patient developed a peri-renal capsule hematoma and another one post-interventional hemoptysis (all four patients were receiving a dual antiplatelet therapy). The estimated bleeding risk for anticoagulated atrial fibrillation patients with a HAS-BLED score between 3 and 4 (3.35 ± 1.0 in the analyzed population) ranged from 3.7 to 8.7%. Figure 2 shows the relative difference between the estimated and observed rates of cerebrovascular events and bleeding complications in our study population.

**Table 2:** Clinical results

	All patients *N* = 189	eGFR≥45 *N* = 116	eGFR< 45 *N* = 73	*p*
Follow-up duration, years	310	188	122	
Cerebrovascular events, n (n/100py)	3 (1.0)	0	3 (2.5)	0.09
• Stroke, n (n/100py)	2	0	2 (1.6)	ns
• TIA, n (n/100py)	1	0	1 (0.8)	ns
Bleeding complications, n (n/100py)	5 (1.6)	0	5 (4.1)	0.06
• Intracranial bleeding	0	0	0	
• Gastrointestinal bleeding	1 (0.3)	0	1 (0.8)	ns
• Other bleeding	4 (1.3)	0	4 (3.3)	ns
All-cause death, n (n/100py)	8 (2.6)	2 (1.1)	6 (4.9)	ns
• Cardiovascular death, n (n/100py)	6 (1.9)	2 (1.1)	4 (3.3)	ns
Periprocedural complications				
• Pericardial effusion, n (%)	0	0	0	
• DRT, n (%)	12 (6.5)	6 (3.2)	6 (8.2)	ns
• Other peri-procedural complications, n (%)	4 (2.1)	2 (1.7)	2 (2.7)	ns

100py = one hundred patient years; TIA = transitory ischemic attack; DRT = device related thrombus

During follow up, six patients died in the subgroup with CKD IIIb or worse (cardiovascular death was diagnosed in four patients) and two other patients in the subgroup with CKD IIIa or better (both with cardiovascular death), see Table 2 for details.

**Fig. 3:**
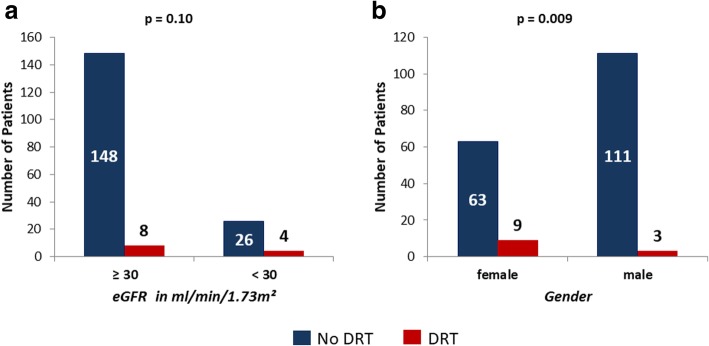
Number of patients with and without device related thrombi (DRT) for: **a**) CKD stage I-IIIb vs. IV-V and **b**) female vs. male

After successful LAA closure DRT were detected in twelve (6.5%) patients (in eleven patients at the first scheduled TEE). Women had significantly more DRT than men (12.5% vs. 2.6%, *p* = 0.009). EGFR in the subgroup of patients with DRT was numerically lower as compared to those without (46.5 vs. 58.3 ml/min/1.73 m^2^, *p* = 0.18). On the other hand, patients with severe impairment of the renal function (eGFR < 30 ml/min/1.73m^2^) showed a clear trend to more likely develop DRT (13.3% vs. 5.1%, *p* = 0.10), as compared to the opposite group, Fig. 3 a and b. All but one patient were on dual antiplatelet therapy at the time point of DRT diagnosis; all DRT patients received a temporary anticoagulation therapy until thrombus resolution or consolidation was confirmed by TEE (at least 6 weeks). Thrombus resolved in ten patients without sequelae, whereas thrombus consolidation (endothelialization) was confirmed in the remaining two patients by serial TEE controls. In these two patients a thromboembolic event was considered very unlikely. Those patients were switched to aspirin monotherapy and showed no change of thrombus size in serial TEE controls. No cardioembolic events were reported in the subgroup of patients with documented DRT.

A residual peri-device leakage was detected by TEE in 61 (32.8%) patients immediately after release of the occluder device, in 50 (26.9%) patients after six weeks and in 39 (21.0%) patients after six months. In all patients residual leakage was < 5 mm and decreased from 2.9 ± 1.1 mm immediately after implantation to 2.5 ± 0.8 mm after six weeks and 2.2 ± 0.9 mm after six months. Patients with detected residual leakage at the index procedure had a significantly lower compression rate (CR), as defined above (see Table 3 for details), while device compression (DC) seemed to have no influence on residual leakage. With decreased number of patients with residual leakage after six weeks and six months, the CR differences between them and the opposite group were no longer significant.

**Table 3:** Device compression and compression rate in patients with peri-device leakage

	post-Implantation			at six weeks			at six months		
	No	Yes	*p*	No	Yes	*p*	No	Yes	*p*
DCmax	1.30 ± 0.14	1.27 ± 0.10	0.21	1,29 ± 0.15	1.30 ± 0.11	0.65	1.29 ± 0.15	1.29 ± 0.10	0.93
DCmin	1.20 ± 0.12	1.20 ± 0.10	0.91	1.19 ± 0.12	1.21 ± 0.11	0.50	1.20 ± 0.13	1.20 ± 0.11	0.93
CRmax	1.50 ± 0.22	1.40 ± 0.18	0.02	1.49 ± 0.23	1.41 ± 0.18	0.12	1.49 ± 0.23	1.41 ± 0.15	0.10
CRmin	1.24 ± 0.18	1.18 ± 0.15	0.07	1.24 ± 0.20	1.20 ± 0.13	0.26	1.23 ± 0.18	1.22 ± 0.15	0.29

DCmax = (nominal device size / minimal device diameter), DCmin = (nominal device size / maximal device diameter) CRmax = (nominal device size / minimal LAA ostium diameter), CRmin = (nominal device size / maximal LAA ostium diameter)

Periprocedural complications occurred in four (2.1%) patients. A temporarily ST-segment elevation on the monitoring electrocardiogram (ECG) presumed air embolism into the coronary arteries in two patients. In both cases immediate angiography revealed no air or obstruction in the coronary arteries and ST-segment elevation resolved spontaneously within a few minutes. In two more patients vascular complications occurred, including a groin hematoma and an artery-venous fistula; the latter had to be treated surgically. No pericardial effusion, device embolization or cerebrovascular events were observed during the implantation procedures.

## Discussion

In this consecutive series of atrial fibrillation patients at moderate or high thromboembolic risk predominated by chronic kidney disease patients, we prospectively demonstrated low rates of cerebrovascular and bleeding events in a long-term follow up after successful LAA closure with the WATCHMAN™ device. Furthermore, the results of our study confirm low complication rates after interventional LAA closure with the WATCHMAN™ device and add important information to the limited data on atrial fibrillation patients with CKD undergoing this procedure.

According to the data of Lip and colleagues, the annual risk of stroke in atrial fibrillation patients increases with the CHA_2_DS_2_Vasc score, whereas the bleeding risk in anticoagulated atrial fibrillation patients increases with the HAS-BLED score [3, 4, 27]. The observed stroke rate in our study is comparable to that reported in the interventional groups of the randomized trials [28] and the same rate in the large series of patients after WATCHMAN™ device implantation included in the EWOLUTION registry [29]. Considering an estimated annual stroke risk between 3.2 and 4.0% based on the CHA_2_DS_2_Vasc score in our study population, the observed stroke rate was 69 to 75% lower than expected. The observed reduction in stroke rate in our study is comparable to that reported for VKA use in atrial fibrillation patients [5], which confirms the efficacy of interventional LAA closure with the WATCHMAN™ device and the non-inferiority of this therapy when compared to VKA use as shown by the randomized trials [20–22]. At the same time, we observed a 56 to 82% reduction in the estimated bleeding rate (the latter calculated on the HAS-BLED score in our study population) [4, 27], which represents the net clinical benefit of interventional LAA closure over VKA use for stroke prevention in atrial fibrillation patients.

The confirmation of the efficacy and safety of LAA closure with the WATCHMAN™ device in a cohort of atrial fibrillation patients, predominated by those with impaired kidney function, should facilitate the decision on the appropriate individual stroke prevention therapy in these patients. Furthermore, our results support the interventional LAA closure in atrial fibrillation patients with absolute contraindication or refusal to oral anticoagulation therapy for stroke prevention. Previous studies reported controversial results regarding stroke risk reduction by VKA therapy in patients with severe CKD [17, 30, 31], while they unanimously reported significantly higher risk of major bleeding. Data on the benefit of NOAC use in these patients are even more limited, though that might be subject to actual investigation. Therefore, the amount of atrial fibrillation patients receiving no anticoagulation therapy for stroke prevention is considered to be higher among those with severe CKD. The results of our study should encourage the interventional LAA closure as an effective and safe alternative stroke prevention therapy in atrial fibrillation patients with severe CKD.

A larger operators’ experience with the device and a thorough preselection of the patients considering the LAA anatomy, as assessed by TEE prior to the implantation procedure, led to a high implantation success rate (98.4%), which was higher than that reported in the randomized trials [20, 32] and comparable to the recently published results on implantation success rate of the WATCHMAN™ device in the large multicentric EWOLUTION registry [29]. Previously, Holmes et al. showed that increasing operator experience is not only related to a higher implantation success but also to lower rates of procedure related serious adverse events [21, 32]. In our study, a low periprocedural complication rate was observed, which is comparable to the reported recent experience with the WATCHMAN™ device [29]. In previously published trials the majority of periprocedural adverse events could be accounted to accidental air embolism and pericardial effusion [20, 21, 29]. While a hemorrhagic pericardial effusion could be avoided in all patients in our study by using TEE guidance for transseptal puncture and with operators’ experience, accidental air embolism in the right coronary artery was observed in two patients despite accurate flushing of the delivery system and transseptal sheath. Both patients had a low left atrial pressure (though 500 ml saline were infused prior to transseptal puncture), which was intermittently negative because of a distinct negative intrathoracic pressure while snoring during deep sedation; the latter was necessary for tolerance of the TEE probe. The use of intracardiac echocardiography for guidance of transseptal puncture and device placement could avoid deep sedation and might be an advantageous alternative to TEE in these patients, especially in those with known sleep apnea.

Concerns are raised by a higher risk for DRT formation in female patients and those with severe CKD [33, 34]. In our study DRT rate was 4.8 times higher in female patients and 2.6 times higher in those with a CKD stage IV or V, as compared to the opposite groups. These findings confirm the importance of systematic TEE controls after the implantation procedure for early DRT detection and safe management of the patients and might justify a more intensive schedule of TEE controls in patients at high risk for DRT formation, such as female patients with severe CKD. Furthermore, a temporarily oral VKA therapy as used in the randomized trials (if not contraindicated) might lower the risk of DRT formation in the pre-endothelialization phase, which in CKD patients is presumably prolonged. These considerations as well as a potential clopidogrel resistance, which might play an important role in DRT formation as previously reported by Ketterer and colleagues [35], should be subject to further investigation.

Residual leakage between the occluder device and the LAA wall is a common finding immediately after the implantation procedure of a WATCHMAN™ device [36]. However, in most cases a residual leakage diminishes with complete endothelialization of the device and according to actual data residual leaks < 5 mm are not associated with increased rates of thromboembolic complications [37]. In our study, maximum and minimal compression rates as defined above were significantly lower in patients with residual peri-device leaks. In this context, choosing the correct implantation strategy and appropriate device size in relation to the LAA ostium morphology and diameter should avoid large residual peri-device leaks. A special LAA anatomy with a proximal arborization into two or more lobes might necessitate the implantation of more than one WATCHMAN™ device, whereas in patients with a residual leak of ≥5 mm percutaneous closure using a vascular plug device might be individually considered [38].

### Limitations

This is not a randomized trial and therefore prone to related bias. Furthermore, the proportion of patients with an eGFR below 60 and 30 ml/min/1.73m^2^ were only 55.6 and 38.6%, respectively. In this study of mostly patients with high bleeding risk or bleeding history we restricted the antithrombotic medication to antiplatelet therapy alone avoiding the initial recommendation for anticoagulation therapy by the manufacturer, except for temporarily anticoagulation in patients with observed DRT at TEE controls, which might have influenced the DRT rates. Collection of clinical data by phone-contact at the last follow-up is related to subjective or mnestic bias which might have influenced the results of our study.

## Conclusions

Atrial fibrillation patients with CKD and interventional LAA closure using the WATCHMAN™ device have low rates of thromboembolic cerebrovascular events or bleeding complications. The risk of DRT formation is higher in female and patients with severe CKD. A temporarily oral anticoagulation therapy (if not contraindicated) and/or shorter time intervals for TEE controls after the implantation procedure might be justified for prevention, early DRT detection and safe management of these patients. A low compression rate, defined as the ratio between the nominal device size and LAA ostium diameter, is predictive for the presence of residual peri-device leaks immediately after the implantation procedure.

## Abbreviations

CKD: Chronic kidney disease
CKD-EPI: Chronic kidney disease epidemiology collaboration
CR: Compression rate
CR_max_: Maximum compression rate
CR_min_: Minimal compression rate
DC: Device compression
DC_max_: Maximum device compression
DC_min_: Minimal device compression
DRT: Device related thrombi
ECG: Electrocardiogram
eGFR: Estimated glomerular filtration rate
KDIGO: Kidney disease improving global outcomes
LAA: Left atrial appendage
NOAC: Non-vitamin-K antagonist oral anticoagulant
NVAF: Non-valvular atrial fibrillation
OAC: Oral anticoagulation
SD: Standard deviation
TEE: Transesophageal echocardiography
VKA: Vitamin K antagonists
